# Planned delivery or expectant management for late preterm pre-eclampsia in low-income and middle-income countries (CRADLE-4): a multicentre, open-label, randomised controlled trial

**DOI:** 10.1016/S0140-6736(23)00688-8

**Published:** 2023-07-29

**Authors:** Alice Beardmore-Gray, Nicola Vousden, Paul T Seed, Bellington Vwalika, Sebastian Chinkoyo, Victor Sichone, Alexander B Kawimbe, Umesh Charantimath, Geetanjali Katageri, Mrutyunjaya B Bellad, Laxmikant Lokare, Kasturi Donimath, Shailaja Bidri, Shivaprasad Goudar, Jane Sandall, Lucy C Chappell, Andrew H Shennan, CRADLE-4 Study Group, Mercy Kopeka, Josephine Miti, Christine Jere, Chipo Hamweemba, Sandra Mubiana, Louise Ntamba Mukosa, Aaron Tembo, Philip Gondwe, Ashalata Mallapur, Umesh Ramadurg, Sahaja Kittur, Prakash Wari, Muttu R Gudadinni, Sangamesh Methapati, Siddu Charki, Rachael Hunter

**Affiliations:** a Department of Women and Children's Health, School of Life Course Sciences, King's College London, London, UK; b Department of Obstetrics and Gynaecology, University of Zambia, Lusaka, Zambia; c Department of Obstetrics and Gynaecology, Ndola Teaching Hospital, Ndola, Zambia; d Department of Obstetrics and Gynaecology, Kitwe Teaching Hospital, Kitwe, Zambia; e Department of Obstetrics and Gynaecology, Kabwe General Hospital, Kabwe, Zambia; f Women's and Children's Health Research Unit, KLE Academy of Higher Education and Research, J N Medical College, Belagavi, Karnataka, India; g S Nijalingappa Medical College and Hangal Shri Kumareshwar Hospital and Research Centre, Bagalkot, Karnataka, India; h Karnataka Institute of Medical Sciences, Hubballi, Karnataka, India; i Bijapur Lingayat District Educational Association (Deemed to be University), Shri B M Patil Medical College Hospital and Research Centre, Bijapur, India

## Abstract

**Background:**

Pre-eclampsia is a leading cause of maternal and perinatal mortality. Evidence regarding interventions in a low-income or middle-income setting is scarce. We aimed to evaluate whether planned delivery between 34^+ 0^ and 36^+ 6^ weeks’ gestation can reduce maternal mortality and morbidity without increasing perinatal complications in India and Zambia.

**Methods:**

In this parallel-group, multicentre, open-label, randomised controlled trial, we compared planned delivery versus expectant management in women with pre-eclampsia from 34^+ 0^ to 36^+ 6^ weeks’ gestation. Participants were recruited from nine hospitals and referral facilities in India and Zambia and randomly assigned to planned delivery or expectant management in a 1:1 ratio by a secure web-based randomisation facility hosted by MedSciNet. Randomisation was stratified by centre and minimised by parity, single-fetus pregnancy or multi-fetal pregnancy, and gestational age. The primary maternal outcome was a composite of maternal mortality or morbidity with a superiority hypothesis. The primary perinatal outcome was a composite of one or more of: stillbirth, neonatal death, or neonatal unit admission of more than 48 h with a non-inferiority hypothesis (margin of 10% difference). Analyses were by intention to treat, with an additional per-protocol analysis for the perinatal outcome. The trial was prospectively registered with ISRCTN, 10672137. The trial is closed to recruitment and all follow-up has been completed.

**Findings:**

Between Dec 19, 2019, and March 31, 2022, 565 women were enrolled. 284 women (282 women and 301 babies analysed) were allocated to planned delivery and 281 women (280 women and 300 babies analysed) were allocated to expectant management. The incidence of the primary maternal outcome was not significantly different in the planned delivery group (154 [55%]) compared with the expectant management group (168 [60%]; adjusted risk ratio [RR] 0·91, 95% CI 0·79 to 1·05). The incidence of the primary perinatal outcome by intention to treat was non-inferior in the planned delivery group (58 [19%]) compared with the expectant management group (67 [22%]; adjusted risk difference –3·39%, 90% CI –8·67 to 1·90; non-inferiority p<0·0001). The results from the per-protocol analysis were similar. There was a significant reduction in severe maternal hypertension (adjusted RR 0·83, 95% CI 0·70 to 0·99) and stillbirth (0·25, 0·07 to 0·87) associated with planned delivery. There were 12 serious adverse events in the planned delivery group and 21 in the expectant management group.

**Interpretation:**

Clinicians can safely offer planned delivery to women with late preterm pre-eclampsia, in a low-income or middle-income country. Planned delivery reduces stillbirth, with no increase in neonatal unit admissions or neonatal morbidity and reduces the risk of severe maternal hypertension. Planned delivery from 34 weeks’ gestation should therefore be considered as an intervention to reduce pre-eclampsia associated mortality and morbidity in these settings.

**Funding:**

UK Medical Research Council and Indian Department of Biotechnology.

## Introduction

810 women have been reported to die every day from preventable causes related to pregnancy and childbirth. 94% of these deaths occur in low-income countries (LICs) and lower-middle-income countries (LMICs).^1^ In particular, women living in sub-Saharan Africa and south Asia have a disproportionately high risk of death.^1^ Hypertensive disorders of pregnancy are a leading cause of maternal death, with pre-eclampsia representing the most serious of these disorders. Pre-eclampsia complicates around 3–5% of pregnancies^2^ and is estimated to cause at least 42 000 maternal deaths^2^ and 500 000 perinatal deaths, including 200 000 stillbirths,^3^ every year. Pre-eclampsia is typically defined as new onset hypertension after 20 weeks’ gestation with evidence of one or more of proteinuria, maternal organ dysfunction, or uteroplacental insufficiency.^4^ Pre-eclampsia can lead to severe consequences for both the woman and infant, including eclampsia, maternal death, and stillbirth. The clinical course is progressive and difficult to predict, with delivery the only curative treatment. Early detection and timely delivery reduce complications for the woman.^5^, ^6^, ^7^ The timing of delivery must consider the risks or benefits of preterm birth for the infant. WHO recommends delivery at 37 weeks’ gestation for all women with pre-eclampsia irrespective of disease severity.^8^ Before 34 weeks, expectant management is considered preferable due to the neonatal risks associated with early preterm birth, with delivery only recommended for severe maternal or fetal compromise.^8^, ^9^ Between 34 and 37 weeks of pregnancy, the optimal timing of delivery is less clear. 2019 evidence from high-income settings has shown maternal benefit associated with planned delivery during this gestation period, with an increase in neonatal unit admissions (compared with expectant management) but no increase in neonatal morbidity.^7^ Fetal death is rare at late preterm gestations in high-income settings, with none reported in a 2022 meta-analysis.^10^ On the basis of our literature search, no published studies to date have reported a comparison of planned delivery versus expectant management for late preterm pre-eclampsia in a LIC or LMIC, despite the overwhelming proportion of maternal and perinatal mortality occurring in these settings. The potential risks and benefits of late preterm delivery for the infant in a low resource setting with varying levels of antenatal, intrapartum, and neonatal care available are likely to be different to those in a high-income setting, and therefore this intervention requires careful evaluation. The aim of this trial was to evaluate whether planned delivery between 34 and 37 weeks’ gestation, in women with pre-eclampsia without an indication for immediate delivery, could reduce adverse pregnancy outcomes, compared with usual care (expectant management), in sites across India and Zambia.

**Research in context**

**Evidence before this study**

A Cochrane Review published in 2017 that compared planned delivery with expectant management for hypertensive disorders from 34 weeks’ gestation to term found that planned delivery was associated with lower maternal mortality and morbidity, but there was insufficient information to draw any conclusions about the effect on the baby. The authors of this review highlighted the need for an individual participant data meta-analysis to better delineate the effect of planned delivery in different types of hypertensive disorders in pregnancy. In 2022, some of the present authors published an individual participant data meta-analysis (IPDMA) comparing planned delivery with expectant management in pre-eclampsia from 34 weeks’ gestation onwards, building on a previous IPDMA that assessed all hypertensive disorders of pregnancy together. We did an electronic search of the Cochrane Central Register of Controlled Trials, PubMed, MEDLINE, and ClinicalTrials.gov, to review the available evidence on timing of delivery in late preterm pre-eclampsia. We used the search terms “pre-eclampsia” OR “preeclampsia” AND “delivery” OR “birth” with the limits “human” and “randomised controlled trial”. We did not restrict our search by language. Cluster randomised trials or studies with quasi-randomised design were excluded, as were trials published before the year 2000. The final search date was Dec 18, 2021. Six trials that compared planned delivery with expectant management in women with pre-eclampsia from 34 weeks’ gestation onward were eligible for inclusion in this IPDMA. Most were assessed as being at low risk of bias. Using one-stage IPD meta-analysis of 1790 participants from these six trials, we found that planned delivery from 34 week's gestation onward significantly reduced the risk of maternal morbidity (adjusted risk ratio [RR] 0·59, 95% CI 0·36–0·98) compared with expectant management. The primary composite perinatal outcome was increased by planned delivery (1·22, 1·01–1·47), driven by short-term neonatal respiratory morbidity. However, infants in the expectant management group were more likely to be born small for their gestational age (RR 0·74, 95% CI 0·55–0·99). All these trials took place in a high-income setting.

**Added value of this study**

The CRADLE-4 trial addresses a key gap in the current evidence around the effect of planned delivery in late preterm pre-eclampsia in low-income or lower-middle-income countries. These countries bear the highest burden of pre-eclampsia-related mortality and morbidity, and it is therefore essential that any interventions targeted at reducing these adverse outcomes are evaluated in the environments where they are most needed. We have shown that, in line with current evidence, planned delivery reduces severe maternal hypertension and other serious complications such as eclampsia and placental abruption. Although our study did not show a significant reduction in the maternal composite outcome, almost all outcomes for the woman favoured planned delivery, with the remaining outcomes showing no difference. The intervention did not increase operative delivery, and length of stay in hospital for the woman was shorter, consistent with findings from previous studies. We found that planned delivery significantly reduced stillbirth, driven by a large difference in antepartum stillbirth (none in the planned delivery group *vs* ten in the expectant management group). This is a novel, and important finding. Previous studies done in high-income countries with very low rates of perinatal mortality have not been able to show perinatal benefit associated with planned delivery. Furthermore, our results show that babies born at late preterm gestations do not have high rates of morbidity, even in settings where neonatal care might be less advanced; this might be due, in part, to the availability of antenatal corticosteroids and kangaroo mother care. Neonatal outcomes were similar between the two management groups, with no significant differences in respiratory outcomes or other important markers of neonatal morbidity such as jaundice or hypoxic ischaemic encephalopathy.

**Implications of all the available evidence**

Our findings, alongside evidence from randomised controlled trials done in high-income countries, support initiating delivery in pre-eclampsia from 34 weeks’ gestation for maternal benefit. Importantly, we have shown that this can be offered without harm to the baby, showing non-inferiority of planned delivery compared with expectant management for our primary perinatal outcome. We provide new evidence showing benefit and safety for the baby, even in settings with variable resource availability. We have shown that in low-income or lower-middle-income settings, planned delivery reduces stillbirth, and should therefore be considered for improving both perinatal and maternal outcomes.

## Methods

### Study design

This was a multicentre, open-label, randomised controlled trial with individual randomisation, across nine sites in India and Zambia, which are currently classified as a lower-middle-income country and a low-income country, respectively. The four sites in India were tertiary level urban referral hospitals based in the state of Karnataka. The five sites in Zambia were tertiary level urban referral hospitals based in the Lusaka, Central, and Copperbelt provinces, including their referring health-care facilities, which serve a mixed urban and rural population. A full site listing is shown in the appendix (p 1). Ethical approval was obtained from King's College London (reference numbers HR-19/20-13535), the University of Zambia (UNZA-301/2019), BVV Sangha's S Nijalingappa Medical College (SNMCIEC/1.1 /2019-2020), and the Women's and Children's Health Research Unit, Karnataka Lingayat Education Society Academy of Higher Education and Research (KAHER/IEC/2019-20/D-251119016).

Before designing the protocol for the interventional phase of the trial, some of the present authors did a 6-month feasibility and acceptability study, seeking to understand the barriers and facilitators to our proposed intervention across the trial sites, including the acceptability of the intervention to pregnant women and their supporting relatives.^11^ This study directly informed trial design, enabling us to develop pragmatic methods of diagnosing pre-eclampsia (in accordance with ISSHP recommendations for low resource settings),^4^ identifying gestational age, and defining clinical outcomes suitable for the local context.

### Participants

A pregnant woman of any age was eligible if she had a clinical diagnosis of pre-eclampsia and a gestational age between 34^+0^ and 36^+6^ weeks, as confirmed by a doctor, with a single-fetus pregnancy or multi-fetal pregnancy and at least one viable fetus. Women with any other co-morbidity (including pre-existing hypertension, diabetes, and HIV) or having had a previous caesarean section, or with the fetus in any presentation, were eligible. Women were excluded if a decision had already been made to initiate delivery within the next 48 h, as recommended for pre-eclampsia with severe features. Site research teams sought written consent from eligible women after providing a full verbal and written description of the trial in her preferred language, supplemented by three short video clips when these were available. A full version of the published study protocol is available online.^11^ There were no substantial changes to the published study design, methods, or outcomes after the start of the trial.

### Randomisation and masking

Baseline participant details were entered onto the trial database by local research assistants. Participants were randomly assigned to planned delivery or expectant management in a 1:1 ratio by a secure web-based randomisation facility hosted by MedSciNet. Randomisation was stratified by centre and minimised by parity, single-fetus pregnancy or multi-fetal pregnancy, and gestational age (34^+ 0^ to 34^+ 6^, 35^+ 0^ to 35^+ 6^, 36^+ 0^ to 36^+ 6^). MedSciNet wrote the randomisation programme and held the allocation code. The randomised allocation was generated by the web-based programme (using a tablet computer or other internet-enabled device) and then directly communicated to the woman and her clinical team. Due to the nature of the intervention, masking of clinicians and participants was not possible.

### Procedures

The intervention consisted of initiation of delivery within 48 h of randomisation (48 h was given to enable corticosteroid administration to accelerate fetal lung maturation if necessary). Expectant management comprised usual care, with delivery at 37 weeks’ gestation or sooner if clinically indicated, in accordance with WHO guidelines. Expectant management included both inpatient and outpatient monitoring depending on local capacity, clinical judgement, and the woman's preferences. Use of antenatal corticosteroids was left to the discretion of the clinical team, in line with local guidance. Method of induction, mode of delivery, intrapartum care, and postnatal care followed local clinical practice at each trial site. Outcomes were recorded on the web-based trial database contemporaneously by site research teams up until maternal and infant primary discharge from hospital. Each participant record was cross-checked by the trial co-ordinator and any queries resolved with local site teams with retrospective case-note review if required. The end of the intervention phase was defined by the date when the last participating woman and infant were discharged from hospital, or 42 days after the final participant was recruited (whichever occurred sooner).

### Outcomes

There was one primary maternal outcome and one primary perinatal outcome. The primary maternal outcome was a composite of maternal multi-organ pre-eclampsia-associated morbidity based on miniPIERS outcomes (including maternal death, CNS, cardiorespiratory, haematological, hepatic, renal variables, and placental abruption, listed in full in our trial protocol)^12^ modified to suit our trial environment,^11^, ^12^, ^13^ with the addition of recorded systolic blood pressure of at least 160 mm Hg after randomisation (on any occasion). The primary perinatal outcome was a composite of neonatal death, antenatal or intrapartum stillbirth, or neonatal unit admission of more than 48 h due to neonatal morbidity (as defined by a clinical indication for admission to the neonatal unit according to local site guidelines). Data for every participant was checked by the trial coordinator. Secondary maternal outcomes comprised individual components of the composite primary outcome (miniPIERS outcomes or recorded systolic blood pressure of ≥160 mm Hg), miniPIERS outcomes detected by clinical diagnosis only, onset of labour, need for antihypertensives before delivery, primary indication for delivery, and process outcomes such as length of stay and time from randomisation to initiation of delivery. Secondary perinatal outcomes comprised individual components of the composite outcome, any admission to the neonatal unit, number of nights in each category of care, total number of nights in hospital, birthweight, birthweight centile, birthweight less than tenth or third centile, gestational age at delivery, Apgar score at 5 min after birth, need for respiratory support, need for supplemental oxygen, confirmed diagnosis of sepsis, antibiotics given for possible serious bacterial infection, hypoxic ischaemic encephalopathy (all grades), and respiratory distress syndrome. Research teams undertook standard assessments of safety, with reporting of adverse events and serious adverse events as specified in the trial protocol and following the usual governance procedures for a clinical trial.

### Statistical analysis

Assuming an anticipated composite adverse maternal outcome incidence of 80% in the expectant management group, on the basis of data from the CRADLE-4 feasibility study,^11^ a sample size of 558 women would provide 90% power to detect a 15% relative risk reduction of the primary maternal outcome in the planned delivery group with a two-sided 5% significance level. With an anticipated 10% loss to follow-up, the overall inflated target for recruitment was 620 women. Assuming a composite adverse perinatal outcome incidence of 24%, based on data from the CRADLE-4 feasibility study,^11^ complete data on 480 women would be required for 90% power to exclude a difference against planned delivery of 10% or more (based on a non-inferiority analysis using a one-sided 5% significance test and 90% CI). This estimate was in line with the planned sample size and overall recruitment target. The primary analysis for all maternal outcomes was by intention to treat with participants analysed in the groups to which they were assigned regardless of protocol non-compliance. The primary analysis for all perinatal outcomes was by both intention to treat and per protocol since the hypothesis under examination for these outcomes was non-inferiority. All outcomes were analysed adjusting for minimisation factors at randomisation, which were gestational age at randomisation, twin pregnancy, and parity. Binary outcomes were analysed using log binomial regression models with results presented as adjusted risk ratios (RRs) with associated CIs. Continuous outcomes were analysed using linear regression models with results presented as differences in means with associated CIs. 95% CIs are presented for all primary outcomes and their main components. 99% CIs are presented for secondary outcomes, in order to minimise the risk of a type I error.

For all perinatal outcomes, all infants (single-fetus pregnancy or multiple-fetal pregnancy) were treated separately, adjusting standard errors for clustering by mother.^14^ Prespecified subgroup analyses were done for primary outcomes based on gestational age at randomisation (test for trend), single-fetus versus multi-fetal pregnancy, country, and region (with a region being tertiary centre and referring health-care facilities). To allow for clinical and logistical delays, we did a prespecified sensitivity analysis on the primary outcomes excluding women and infants randomly assigned to the planned delivery group for whom initiation of delivery was more than 96 h post randomisation. Data analyses were done with STATA version 17. An independent data monitoring committee reviewed trial progress and conduct, including all reported serious adverse events, at regular intervals throughout the study. No formal interim analysis was planned, and guidance for early cessation of the trial followed the Haybittle-Peto principle that overwhelming evidence is needed in favour of one treatment option, such that randomisation would no longer be ethical. The trial was prospectively registered with the ISRCTN registry (ISRCTN10672137).

### Role of the funding source

The funders of the study had no role in study design, data collection, data analysis, data interpretation, or writing of the report.

## Results

Between Dec 19, 2019, and March 31, 2022, 881 women were screened, and 584 women were found to be eligible, of whom 565 were enrolled (figure), across four referral sites in India and five referral sites and their linked primary health-care facilities in Zambia (appendix p 1). 284 women were allocated to planned delivery and 281 to expectant management (figure). For the intention-to-treat analysis, data from 282 women and 301 babies in the planned delivery group and 280 women and 300 babies in the expectant management group were included. Follow-up to maternal and infant discharge continued until May 12, 2022. Two women allocated to planned delivery withdrew consent, and one woman was lost to follow-up in the expectant management group (figure). Baseline maternal characteristics appeared balanced between the two groups (table 1). A high proportion of women in each group had their pregnancy dated using the self-reported date of their last menstrual period (122 [43%] in the planned delivery group and 142 [51%] in the expectant management group). Only five (2%) women in the planned delivery group and 15 (5%) women in the expectant management group were prescribed aspirin at any stage during their pregnancy.

**Figure:**
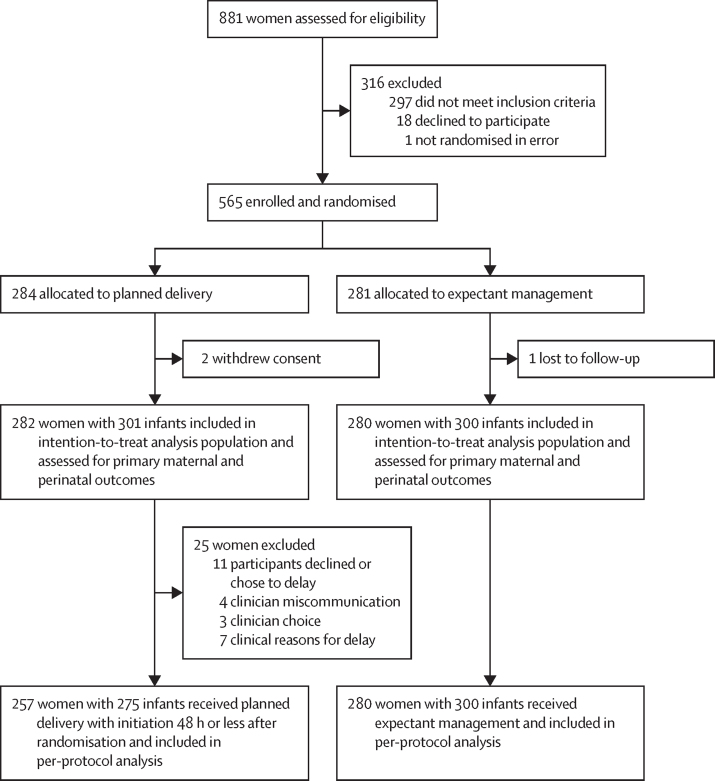
Trial profile

**Table 1:** Baseline maternal characteristics at enrolment

		**Planned delivery (n=282)**	**Expectant management (n=281)**
Maternal age, years		28·53 (6·66)	28·07 (6·32)
Ethnicity			
	Black African	204 (72%)	202 (71·9%)
	Asian Indian	78 (28%)	79 (28·1%)
Educational level			
	None	6 (2%)	4 (1·4%)
	Primary	76 (27%)	70 (24·9%)
	Secondary	159 (56%)	157 (55·9%)
	Tertiary	41 (15%)	50 (17·8%)
No previous births*		110 (39%)	106 (37·7%)
One or more previous birth (≥24 weeks)		172 (61%)	175 (62·3%)
Previous caesarean section		53/172 (31%)	42/175 (24·0%)
High blood pressure in a previous pregnancy			
	No	140/184 (76%)	120/186 (64·5%)
	Yes	37/184 (20%)	51/186 (27·4%)
	Unknown	7/184 (3%)	15/186 (8·1%)
BMI, kg/m^2^		26·9 (5·8)	27·5 (6·1)
First trimester weight recorded		50 (18%)	61 (21·7%)
Any tobacco use		0	0
Pre-existing chronic hypertension		18 (6%)	29 (10·3%)
Pre-existing chronic renal disease		0	0
HIV positive		12 (4%)	12 (4·3%)
Pre-pregnancy diabetes		2 (1%)	2 (0·7%)
Gestational diabetes		3 (1%)	6 (2·1%)
Aspirin prescribed during pregnancy		5 (2%)	15 (5·3%)
Gestational age determination method			
	Last menstrual period	122 (43%)	142 (50·5%)
	Early scan (before 24 weeks)	102 (36%)	96 (34·2%)
	Late scan (at or after 24 weeks)	58 (21%)	43 (15·3%)
Median gestational age, weeks		35·7 (34·9–36·4)	35·6 (34·9–36·3)
Gestational age category*			
	34 to <35 weeks	81 (29%)	78 (27·8%)
	35 to <36 weeks	83 (29%)	90 (32·0%)
	36 to <37 weeks	118 (42%)	113 (40·2%)
Single fetus pregnancy*		263 (93%)	261 (92·9%)
Highest systolic blood pressure leading to pre-eclampsia diagnosis, mm Hg		158·2 (13·9)	157·7 (13·9)
Highest diastolic blood pressure leading to pre-eclampsia diagnosis, mm Hg		103·3 (9·6)	103·0 (9·5)
Severity of systolic hypertension at diagnosis			
	≤149 mm Hg	70 (25%)	80 (28·5%)
	150–159 mm Hg	97 (34%)	76 (27·0%)
	≥160 mm Hg	115 (41%)	125 (44·5%)
Proteinuria at diagnosis (dipstick)			
	1+	120 (43%)	114 (40·6%)
	2+	126 (45%)	121 (43·1%)
	3+	28 (10%)	38 (13·5%)
	4+	8 (3%)	8 (2·8%)

Data are mean (SD), n (%), or median (IQR).

* Minimisation factors used to ensure balance at randomisation.

The proportion of women with the primary maternal outcome (table 2) was lower in the planned delivery group (154 [55%]) compared with the expectant management group (168 [60%]), but the difference was not statistically significant (adjusted RR 0·91, 95% CI 0·79 to 1·05). Planned delivery was associated with a similar incidence of the primary perinatal outcome compared with the expectant management group (58 [19%] in the planned delivery group *vs* 67 [22%] in the expectant management group; adjusted RR 0·88, 95% CI 0·64 to 1·21; table 2). The risk difference was less than 10% (–3·39%, 90% CI –8·67 to 1·90, p value for non-inferiority <0·0001); hence we can conclude non-inferiority of planned delivery compared with expectant management. The per-protocol analysis produced similar findings (adjusted RR 0·88, 95% CI 0·64 to 1·23, non-inferiority risk difference –3·22%, 90% CI –8·61 to 2·18).

**Table 2:** Primary maternal and perinatal outcome

	**Planned delivery (n=282)**	**Expectant management (n=280)**	Risk ratio*(95% CI), p for superiority	Risk difference*(90% CI, p for non-inferiority)
**Primary maternal outcome**				
Intention to treat	154/282 (55%)	168/280 (60%)	0·91 (0·79–1·05), p=0·182	..
**Individual components**				
Post-randomisation severe hypertension	123/282 (44%)	146/280 (52%)	0·83 (0·70–0·99), p=0·035	..
Maternal morbidity and mortality	61/282 (22%)	66/280 (24%)	0·92 (0·68–1·25), p=0·601	..
Maternal morbidity and mortality detected by clinical diagnosis only†	14/282 (5%)	24/280 (9%)	0·58 (0·31–1·09), p=0·091	..
**Primary perinatal outcome**				
Intention to treat	58/301 (19%)	67/300 (22%)	0·88 (0·64–1·21), p=0·441	−3·39% (−8·67 to 1·90), p<0·0001
Per protocol	52/275 (19%)	67/300 (22%)	0·88 (0·64–1·23), p=0·456	−3·22% (−8·61 to 2·18), p<0·0001
**Individual components**				
Stillbirth	3/301 (1%)	12/300 (4%)	0·25 (0·07–0·87), p=0·029	..
Neonatal death‡	7/301 (2%)	5/300 (2%)	..	..
Neonatal unit admission for >48 h	51/301 (17%)	52/300 (18%)	1·00 (0·71–1·41), p=0·994	..

Data are n (%) unless otherwise specified.

* Analysis adjusted for gestational age at randomisation, twin pregnancy, and parity.

† Any one of: maternal death, hepatic haematoma, rupture, Glasgow coma score <13, stroke, cortical blindness, reversible ischaemic neurological deficit, retinal detachment, postpartum haemorrhage requiring transfusion or hysterectomy, placental abruption, myocardial ischaemia or infarction, eclampsia, requiring >50% oxygen for greater than 1 h, severe breathing difficulty, or pulmonary oedema.

‡ Excluding deaths due to congenital anomalies, risk ratio not calculated due to pooled event rate <5% (as per statistical analysis plan for this variable).

Prespecified analysis of individual components of the primary maternal and perinatal composite outcomes showed a significant reduction in post-randomisation severe hypertension in women allocated to planned delivery (adjusted RR 0·83, 95% CI 0·70–0·99), with a reduction in the same direction, which was not statistically significant, seen in the maternal morbidity and mortality component (0·92, 0·68–1·25). We identified a significant reduction in stillbirth associated with planned delivery (0·25, 0·07–0·87), with no significant differences observed in neonatal death (seven [2%] in the planned delivery group *vs* five [2%] in the expectant management group) or neonatal unit admission for more than 48 h (adjusted RR 1·00, 95% CI 0·71–1·41) between the two groups. The reduction in stillbirth was driven by a marked difference in antepartum stillbirths, with none occurring in the planned delivery group and ten occurring in the expectant management group. The number needed to treat for planned delivery to prevent one antepartum stillbirth was 33 (95% CI 18–193).

The prespecified analysis of selected individual components of the maternal morbidity composite did not show significant differences in the proportion of women in the planned delivery group who had eclampsia (adjusted RR 0·50, 99% CI 0·08 to 3·07), placental abruption (0·38, 0·07 to 2·15), and postpartum haemorrhage requiring transfusion or hysterectomy (0·69, 0·20 to 2·40; table 3), although event rates for these clinical endpoints were lower in the planned delivery group. Other secondary descriptive maternal outcomes show that there was one (<1%) maternal death and four (1%) women admitted to the intensive care unit in the planned delivery group, compared with three (1%) maternal deaths and ten (4%) women admitted to the intensive care unit in the expectant management group (table 3, appendix p 2). The majority (264 [99%] of 266) of women allocated to planned delivery had trial allocation documented as their primary indication for delivery. Women allocated to expectant management were most frequently delivered due to reaching 37 weeks’ gestation (81 [34%] of 240), severe maternal symptoms (71 [30%] of 240), and fetal compromise (33 [14%] of 240). The mean time from randomisation to initiation of delivery was 2·37 days (SD 6·06) for women in the planned delivery group, compared with 5·54 days (SD 7·55) for women in the expectant management group. A high proportion of women across both groups received antenatal corticosteroids (168 [60%] in the planned delivery group *vs* 148 [53%] in the expectant management group), with rates of antihypertensive use (275 [98%] *vs* 274 [98%]) and magnesium sulphate administration (81 [29%] *vs* 96 [34%]) also similar between the two groups. The mean length of stay for women allocated to planned delivery (6·38 days, SD 4·75) was significantly lower compared with those allocated to expectant management (8·19 days, SD 5·07; adjusted mean difference –1·81, 99% CI –2·88 to –0·74). The proportion of vaginal deliveries was similar between the two groups (adjusted RR 0·95, 99% CI 0·74 to 1·24; table 4). Secondary perinatal outcomes showed the median gestational age at delivery was 252 days compared with 255 days for babies born to women in the planned delivery group and expectant management group, respectively (table 4). Median infant birthweight in the planned delivery group was 2340 g (IQR 2000 to 2700) and 2300 g (IQR 2000 to 2700) in the expectant management group. Birthweight centile was significantly higher in those with planned delivery (median difference 4·4, 99% CI 0·5 to 8·8), with fewer infants born less than the tenth centile, although this difference was not significant (adjusted RR 0·85, 99% CI 0·64 to 1·13). Proportions of overall neonatal unit admission were similar between the two groups (119 [40%] of 298 in the planned delivery group *vs* 124 [43%] of 288 in the expectant management group), with only four infants (two in each group) requiring acute-level (invasive ventilation) care. Overall, no statistically significant differences in short-term neonatal complications were observed between the two management groups. Markers of respiratory morbidity such as the proportion of infants needing respiratory support (24 [8%] *vs* 24 [8%], adjusted RR 0·98, 99% CI 0·49 to 1·99), supplemental oxygen (43 [14%] *vs* 55 [19%], 0·77, 0·48 to 1·24), or with respiratory distress syndrome (28 [9%] *vs* 29 [10%]) were similar between the two groups, and lower in the planned delivery group. Rates of other secondary perinatal outcomes were also similar (appendix p 4). Mean number of nights in hospital was 4·68 days (SD 4·70) and 5·18 days (SD 5·50) for infants in the planned delivery group and expectant management group, respectively (table 4).

**Table 3:** Secondary maternal outcomes

		**Planned delivery**	**Expectant management**	Effect measure*(99% CI)	**p value**
Eclampsia		3/282 (1%)	6/280 (2%)	aRR 0·50 (0·08 to 3·07)	0·329
Placental abruption		3/282 (1%)	8/280 (3%)	aRR 0·38 (0·07 to 2·15)	0·152
Postpartum haemorrhage requiring transfusion or hysterectomy		7/282 (3%)	10/280 (4%)	aRR 0·69 (0·20 to 2·40)	0·449
Platelet count <50 × 10^9^ per L without blood transfusion		5/238 (2%)	4/250 (2%)	aRR 1·31 (0·24 to 7·27)	0·681
Hepatic dysfunction†		30/171 (18%)	32/179 (18%)	..	..
Acute renal insufficiency†		5/176 (3%)	5/190 (3%)	..	..
Maternal death		1/282 (<1%)	3/280 (1%)	..	..
Maximum systolic blood pressure post-randomisation, mm Hg		158·32 (14·01)	160·46 (15·94)	..	..
Onset of labour					
	Induced	139/282 (49%)	104/280 (37%)	..	..
	Pre-labour caesarean section	127/282 (45%)	136/280 (49%)	..	..
	Spontaneous	16/282 (6%)	38/280 (14%)	..	..
	PROM and augmentation	0/282	2/280 (1%)	..	..
Need for anti-hypertensives before delivery		275/282 (98%)	274/280 (98%)	..	..
Any antenatal corticosteroids		168/282 (60%)	148/280 (53%)	..	..
Complete course received		106/282 (38%)	106/280 (38%)	..	..
Primary indication for delivery‡ (non-exclusive)					
	Trial allocation to planned delivery arm	264/266 (99%)	0/240	..	..
	Reaching 37 weeks' gestation	3/266 (1%)	81/240 (34%)	..	..
	Severe maternal symptoms	4/266 (2%)	71/240 (30%)	..	..
	Fetal compromise on ultrasound	5/266 (2%)	13/240 (5%)	..	..
	Fetal compromise on cardiotocography	1/266 (<1%)	16/240 (7%)	..	..
	Fetal compromise on intermittent auscultation	4/266 (2%)	33/240 (14%)	..	..
	Maternal haematological abnormality	0/266	3/240 (1%)	..	..
	Maternal biochemical abnormality	0/266	8/240 (3%)	..	..
	Maternal hypertension not controlled by maximal therapy	4/266 (2%)	30/240 (13%)	..	..
	Intrauterine fetal death	0/266	6/240 (3%)	..	..
	Other	1/266 (<1%)	10/240 (4%)	..	..
Process outcomes					
	Time from randomisation to initiation of delivery, days	2·37 (6·06)	5·54 (7·55)	MD −3·18 (−4·63 to −1·72)	<0·0001
	Time from randomisation to delivery, days	3·01 (6·06)	5·89 (7·59)	MD −2·88 (−4·34 to −1·42)	<0·0001
	Length of stay, days	6·38 (4·75)	8·19 (5·07)	MD −1·81 (−2·88 to −0·74)	<0·0001

Data are n (%) or mean (SD) unless otherwise specified. aRR=adjusted risk ratio. MD=mean difference. PROM=pre-labour rupture of membranes.

* Risk ratios are adjusted for gestational age at randomisation (34 weeks, 35 weeks, or 36 weeks), parity (multiparous *vs* primiparous), and multifetal pregnancy.

† Not tested due to missing data >20% in both groups.

‡ Excluding women who went into spontaneous labour.

**Table 4:** Secondary perinatal outcomes

		**Planned delivery**	**Expectant management**	Effect measure*(99% CI)	**p value**
Stillbirth					
	Antepartum stillbirth	0/301	10/300 (3%)	..	..
	Intrapartum stillbirth	3/301 (1%)	2/300 (1%)	..	..
Gestation at birth, days		252 (246 to 257), n=301	255 (248 to 259), n=300	MedD −3·0 (−4·0 to −1·0)	<0·0001
Gestation at birth					
	34 to <35 weeks	58/301 (19%)	30/300 (10%)	..	..
	35 to <36 weeks	78/301 (26%)	82/300 (27%)	..	..
	36 to <37 weeks	123/301 (41%)	88/300 (29%)	..	..
	≥37 weeks	42/301 (14%)	100/300 (33%)	..	..
Vaginal birth		115/301 (38%)	119/300 (40%)	aRR 0·95 (0·74 to 1·24)	0·650
Birthweight, g		2340 (2000 to 2700), n=301	2300 (2000 to 2700), n=300	..	..
Birthweight centile†		22·8 (7·7 to 55·8), n=301	16·9 (3·8 to 41·9), n=300	MedD 4·4 (0·5 to 8·8)	0·003
Small-for-gestational age (<10th centile)†		97/301 (32%)	115/300 (38%)	aRR 0·85 (0·64 to 1·13)	0·137
Small-for-gestational age (<3rd centile)†		35/301 (12%)	64/300 (21%)	..	..
Apgar score at 5 min		9·0 (8·0 to 9·0), n=298	9·0 (8·0 to 9·0), n=288	MedD 0·0 (0·0 to 0·0)	0·178
Need for resuscitation		36/298 (12%)	45/288 (16%)	aRR 0·78 (0·46 to 1·33)	0·227
Any admission to neonatal unit		119/298 (40%)	124/288 (43%)	aRR 0·97 (0·77 to 1·24)	0·784
Number of nights in neonatal unit		3·63 (4·58), n=119	4·15 (5·15), n=124	MD −0·53 (−2·21 to 1·15)	0·412
Number of nights in each level of care‡					
	Acute care	7·50 (6·36), n=2	1·50 (0·71), n=2	..	..
	Subacute care	4·68 (4·44), n=90	4·91 (5·25), n=104	..	..
	Kangaroo mother care	4·68 (3·31), n=41	4·48 (3·66), n=42	..	..
	Normal care	3·15 (1·98), n=243	3·37 (2·61), n=234	..	..
Total number of nights in hospital		4·68 (4·70), n=298	5·18 (5·50), n=288	..	..
Need for respiratory support		24/298 (8%)	24/288 (8%)	aRR 0·98 (0·49 to 1·99)	0·949
	Endotracheal ventilation	2/298 (1%)	2/288 (1%)	..	..
	Continuous positive airways pressure	23/298 (8%)	24/288 (8%)	..	..
Need for supplemental oxygen		43/298 (14%)	55/288 (19%)	aRR 0·77 (0·48 to 1·24)	0·157
Confirmed diagnosis of sepsis§		1/298 (<1%)	1/288 (<1%)	..	..
Antibiotics for possible serious bacterial infection		35/298 (12%)	34/288 (12%)	..	..
Hypoxic ischaemic encephalopathy		14/298 (5%)	14/288 (5%)	..	..
Respiratory distress syndrome		28/298 (9%)	29/288 (10%)	..	..

Data are n (%); mean (SD), n; or median (IQR), n. aRR=adjusted risk ratio. MedD=median difference.

* Risk ratios are adjusted for gestational age at randomisation (34 weeks, 35 weeks, or 36 weeks), parity (multiparous *vs* primiparous), and multifetal pregnancy. Median differences are unadjusted.

† Calculated using intergrowth centiles.

‡ Fetuses might have received more than one level of care, including normal care on the postnatal ward.

§ Positive blood cultures.

There was a total of 33 serious adverse events (affecting 32 pregnancies) during the trial (appendix p 6). The events comprised four maternal deaths (one in the planned delivery group compared with three in the expectant management group); 14 neonatal deaths (eight in the planned delivery group compared with six in the expectant management group), which included two linked to congenital anomalies; and 15 stillbirths (three in the planned delivery group compared with 12 in the expectant management group). None of these serious adverse events were deemed to be unexpected or related to the intervention.

In the prespecified subgroup analyses (unpowered), we found no significant interaction between the incidence of the primary maternal or perinatal outcome and gestational age at randomisation, single-fetus or multifetal pregnancy, country, or region (appendix p 9). A prespecified sensitivity analysis excluding women or infants randomly allocated to the planned delivery group with initiation of delivery after 96 h did not alter our findings in any way (appendix p 8).

## Discussion

In this randomised controlled trial of planned delivery versus expectant management for women with late preterm pre-eclampsia in India and Zambia, we showed that planned delivery significantly reduces severe maternal hypertension, with an important but non-significant reduction in maternal morbidity and mortality. For the fetus or infant, we found that planned delivery did not increase perinatal mortality or morbidity, and significantly reduced the risk of stillbirth, particularly for those in the antenatal period. Secondary maternal and perinatal outcomes were consistent with our main findings, showing fewer short-term maternal complications with no difference in short-term neonatal complications. Overall, best estimates of these secondary treatment effects were in the direction favouring planned delivery, with no indication of harm to the fetus or infant. Planned delivery did not increase rates of operative delivery and was associated with a significant reduction in maternal hospital stay and equivalent neonatal hospital stay.

To our knowledge, this trial is the first to be published evaluating optimal timing of delivery in pre-eclampsia between 34^+ 0^ and 36^+ 6^ weeks’ gestation in LICs and LMICs and is strengthened by its relevance to settings where the vast burden of pre-eclampsia-related morbidity and mortality exists. The inclusion of two different countries with different health-care systems and populations adds to the potential applicability of our results. Reassuringly, the proportion of infants requiring neonatal unit stay, respiratory interventions, or with neonatal morbidity was not increased by the intervention, suggesting planned delivery can be safely implemented in countries with less neonatal resources. Our trial sites incorporated tertiary level hospitals and their local network of primary level health-care facilities, serving a mixed urban and rural population, in accordance with national referral pathways. Therefore, we anticipate our findings would apply to women across different geographical contexts. Our low loss to follow-up rate (one participant) and low rate of missing data, alongside robust in-country oversight from the trial coordinator, provides confidence in the quality and completeness of our data.

A 2021 trial^15^ evaluating therapeutic hypothermia for moderate and severe neonatal encephalopathy, an intervention that has been proven to work in a high-income setting, has shown that such interventions might have a different effect in a low-resource setting. These results highlight the importance of generating evidence from LICs and LMICs before implementing interventions, and the importance of gaining a thorough understanding of the trial environment. The varied disease phenotypes in different populations and settings might also provide new insights into the efficacy of interventions. Our trial was done in settings with variable resource availability, shown by monthly site audits highlighting differences in access to blood pressure monitors, urinalysis sticks, laboratory reagents, and neonatal unit equipment between sites, with rural health-care facilities often not having these key resources. The 6-month feasibility and acceptability study that preceded the interventional phase of the trial enabled us to design a pragmatic protocol and analysis plan, suited to the context, which strengthened our engagement with local health-care partners, the consent process, and our ability to screen and enrol the target number of participants; it also enabled accurate detection of clinical outcomes and adaptation of definitions where necessary. This initial phase enhanced our successful delivery of the trial despite the challenges of working in these settings and, more broadly, the COVID-19 pandemic. However, a larger sample size might have enabled identification of a statistically significant reduction in adverse maternal outcomes, associated with planned delivery, as seen in studies across high-income settings. The planned delivery group had a lower proportion of babies with the primary perinatal outcome, despite a lower than anticipated event rate in the expectant management group. There was no evidence of harm to the infant, which supports our conclusion that planned delivery can be safely recommended. Although there were fewer serious adverse events in the planned delivery group compared with the expectant management group, the high number of serious adverse events overall shows the unacceptably high levels of maternal and perinatal mortality in these settings.

A further challenge during the trial was reaching women with late preterm pre-eclampsia before they developed severe features of the disease. Delays in detection, diagnosis, and referral across local sites meant it was sometimes difficult for site research teams to reach these women at an earlier stage in their disease and could partly explain the smaller than anticipated difference in maternal outcomes between the two groups. Additionally, the small mean difference in time from randomisation to initiation of delivery between the two groups highlights the rapidly progressive and unpredictable nature of pre-eclampsia, particularly in these settings, such that women allocated to expectant management frequently deteriorated and required delivery before 37 weeks’ gestation. This narrow time difference between the groups, which is similar to that found in other studies,^7^, ^10^ could also explain the absence of a statistically significant difference in overall maternal outcomes between the two groups. Importantly, other clinical outcomes such as postpartum haemorrhage or operative delivery were not increased in the planned delivery group, indicating no additional harm to the woman associated with the intervention.

The PHOENIX trial^7^ compared planned delivery with expectant management for pre-eclampsia between 34^+ 0^ and 36^+ 6^ weeks’ gestation and was done in a high-income setting. This trial was the largest reported study to date, and found that planned delivery significantly reduced adverse maternal outcomes but increased the primary perinatal outcome of neonatal unit admission. Overall, the prevalence of serious adverse outcomes in this setting was rare. When incorporated into a larger individual participant data meta-analysis (IPDMA),^10^ combining data from six randomised controlled trials that evaluated planned delivery from 34 weeks’ gestation onwards, these findings remained consistent, with the results of this IPDMA showing a significant reduction in adverse maternal outcomes associated with planned delivery from 34 weeks’ gestation, but an increase in short-term neonatal complications, primarily respiratory distress syndrome. These findings might in part be explained by the wide variation in antenatal corticosteroid use observed in these trials, with those studies done later in the observed period showing greater antenatal corticosteroid use, and no difference in respiratory morbidity between management groups. The high rates of antenatal corticosteroid use in our CRADLE-4 trial show that this intervention is widely available even in lower-resource settings and might partly explain the similar neonatal outcomes observed in both management groups. Although use of antenatal corticosteroids beyond 34 weeks requires further evaluation,^16^ the recently published ACTION-I trial showed that antenatal dexamethasone for women in low-resource countries at risk of preterm birth significantly reduced the risk of neonatal death or stillbirth, with no increase in the incidence of possible maternal bacterial infection.^17^ The CRADLE-4 trial fills a crucial knowledge gap in the evidence relating to timing of delivery, with none of these previous studies evaluating the intervention in an LIC or LMIC. Our findings are consistent with current evidence and supported by a clear biological rationale; planned delivery is well established to provide maternal benefit in the context of pre-eclampsia,^6^ and is associated with higher rates of vaginal delivery, as shown in a 2019 trial^7^ and 2022 meta-analysis.^10^ The significant reduction in severe maternal hypertension observed with planned delivery in this trial is likely to be of clinical benefit, since we know that severe hypertension is associated with an increased risk of adverse maternal outcomes.^18^

In contrast to previous studies, we have shown that planned delivery between 34^+ 0^ and 36^+ 6^ weeks’ gestation for pre-eclampsia in an LIC or LMIC does not increase harm compared with expectant management, but also significantly reduces the risk of stillbirth, with no increase in short-term neonatal complications or neonatal death. In the recently published IPDMA^10^ comparing planned delivery with expectant management in late preterm pre-eclampsia in high-income settings, there were no stillbirths. In the CRADLE-4 trial, 15 women (2·7%) had a stillborn child. This difference highlights the context in which we evaluated planned delivery, and the high rates of pre-eclampsia-associated perinatal mortality that occur in settings with fragile health-care systems and limited resources. An estimated 2·6 million stillbirths occur every year, 98% of which are in LICs or LMICs,^3^ with extensive psychological, physical, and economic consequences.^19^ The number needed to treat to prevent one stillbirth in our trial was 33, considerably lower than the 554 needed to treat^20^ to prevent one stillbirth via post-dates induction of labour in the UK; clinicians and women might therefore feel there is sufficient rationale to offer planned delivery to women with pre-eclampsia from 34 weeks’ gestation onwards. Despite often limited neonatal unit resources, we have shown that in pre-eclampsia after 34 weeks’ gestation, delivery offers clinical benefit to both the infant and the woman. Our secondary perinatal outcomes provide reassuring evidence to support this finding, showing low rates of neonatal complications overall and no difference in neonatal unit admissions or length of stay between the two groups. Supporting a policy of planned delivery, we found a reduction in the proportion of infants born small for gestational age in the planned delivery group, with similar birthweights in each group. These results are consistent with a similar intervention for infants with suspected intrauterine growth restriction,^21^ which found, at 2 years of age, that normal birthweight (increased with planned delivery) increased the chance of a normal neurodevelopmental score.^22^ 2-year follow-up of infants in the PHOENIX trial showed that neurodevelopmental scores were within the normal range for infants in both management groups,^23^ consistent with 2-year and 5-year follow-up of infants in the HYPITAT-II trial which found no significant differences at 5 years of age between infants in the planned delivery and expectant management groups.^24^, ^25^

A formal health-care resource use analysis will be published separately, alongside qualitative data exploring women's experiences of participating in the trial; however, the process outcomes presented here such as length of stay and level of neonatal care required would suggest that planned delivery might be cost-saving for the health-care system, consistent with the cost savings for a high-income setting reported by the PHOENIX trial.^7^, ^26^

These findings have important implications for health-care professionals working in LICs and LMICs, and for women who develop pre-eclampsia. Given the strong body of evidence to support planned delivery from 34 weeks’ gestation for maternal benefit, combined with the new findings from this trial showing both infant safety and a reduction in the risk of stillbirth, we conclude that clinicians can safely offer planned early birth to women with late preterm pre-eclampsia, even without severe features, in an LIC or LMIC, from 34 weeks’ gestation onwards.

Further research must focus on identifying local barriers and facilitators to implementation, engaging communities to raise awareness of pre-eclampsia, and understanding the social and economic factors that might influence a woman's decision to seek antenatal care as well as the wider determinants of the health-care system and its ability to provide safe, timely, and good quality care. This research should include accurate gestational age determination and precise diagnosis of pre-eclampsia. We anticipate that our findings will be incorporated into national and international guidance on timing of delivery in pre-eclampsia, as supported by a policy lab focused on implementation strategies, which indicated positive engagement and commitment from key stakeholders. Context matters: we have shown that even in low resource settings, planned delivery can be safely and effectively implemented, and is recommended to reduce adverse pregnancy outcomes in late preterm pre-eclampsia, particularly stillbirth. This intervention should form part of a concerted global effort to end all maternal and perinatal deaths from preventable causes.

## Data sharing

The dataset will be available to appropriate academic parties on request to the chief investigator (AHS) in accordance with the data sharing policies of King's College London, with input from the co-investigator group where applicable.

## Declaration of interests

JS is a National Institute for Health Research (NIHR) Senior Investigator and is supported by the NIHR Applied Research Collaboration South London at King's College Hospital NHS Foundation Trust. All other authors declare no competing interests.
