# Supplementary material for: Planned delivery or expectant management for late preterm pre-eclampsia in low-income and middle-income countries (CRADLE-4): a multicentre, open-label, randomised controlled trial
Source: Lancet. 2023 Jul 29;402(10399):386–96. doi: 10.1016/S0140-6736(23)00688-8 (PMC11667733; doi:10.1016/S0140-6736(23)00688-8)

THE LANCET

Supplementary appendix

This appendix formed part of the original submission and has been peer reviewed. We post it as supplied by the authors.

Supplement to: Beardmore-Gray A, Vousden N, Seed PT, et al. Planned delivery or expectant management for late preterm pre-eclampsia in low-income and middle-income countries (CRADLE-4): a multicentre, open-label, randomised controlled trial. *Lancet* 2023; published online June 29. https://doi.org/10.1016/S0140-6736(23)00688-8.

Appendix

Supplementary Table S1 Recruitment by Main Site

Site	Planned delivery n=282	Expectant management n=281
**Country**		
Zambia	205 (72·7%)	202 (71·9%)
India	77 (27·3%)	79 (28·1%)
**Zambia (city)**		
Kabwe	31 (11·0%)	29 (10·3%)
Kitwe	31 (11·0%)	30 (10·7%)
Lusaka	85 (30·1%)	88 (31·3%)
Ndola	58 (20·6%)	55 (19·6%)
**Lusaka (centre[s])**		
University Teaching Hospital	24 (8·5%)	26 (9·3%)
Levy Mwanawasa Teaching Hospital	14 (5·0%)	14 (5·0%)
Kanyama 1st level hospital	17 (6·0%)	14 (5·0%)
Chipata 1st level hospital	10 (3·5%)	13 (4·6%)
Chilenje 1st level hospital	6 (2·1%)	6 (2·1%)
Chawama 1st level hospital	4 (1·45)	2 (0·7%)
Matero 1st level hospital	10 (3·5%)	13 (4·6%)
**Ndola (centre[s])**		
Ndola Teaching Hospital	58 (20·6%)	55 (19·6%)
**Kabwe (centre[s])**		
Kabwe Teaching Hospital	31 (11·0%)	28 (10·0%)
Mine Hospital	0	1 (0·4%)
**Kitwe (centre[s])**		
Kitwe Teaching Hospital	31 (11·0%)	30 (10·7%)
**India (city and centre)**		
Belgaum (Jawaharlal Nehru Medical College)	8 (2·8%)	14 (4·3%)
Bagalkot (S. Nijalingappa Medical College and Hangal Shri Kumareshwar Hospital and Research Centre)	3 (1·1%)	3 (1·1%)
Hubballi (Karnataka Institute of Medical Sciences Hubballi)	59 (20·9%)	59 (21·0%)
Vijaypura (BLDE [Deemed to be University] Shri B. M. Patil Medical College Hospital and Research Centre)	7 (2·5%)	5 (1·8%)

Data are n (%), mean (SD), or median (IQR).

Supplementary Table S2 Baseline enrolment characteristics and secondary descriptive maternal outcomes

	**Planned delivery n=282**	**Expectant management n=281**
**Baseline characteristics at enrolment**		
**Previous pregnancy**	184 (65·2%)	186 (66·2%)
**High blood pressure in a previous pregnancy?**	37 (13·1%)	51 (18·1%)
**If history of high blood pressure in previous pregnancy, was there a diagnosis of:**		
Pre-eclampsia	20 (7·1%)	28 (10·0%)
Unsure	16 (5·7%)	20 (7·1%)
Eclampsia	1 (0·4%)	3 (1·1%)
**Tobacco use**	0	0
**Aspirin taken during this pregnancy**	5 (1·8%)	15 (15·3%)
Started in 1st trimester	1 (0·4%)	9 (3·2%)
Started in 2nd trimester	4 (1·4%)	5 (1·8%)
Started in 3rd trimester	0 (0·0%)	1 (0·4%)
**Secondary maternal outcomes**	**n=282**	**n=280**
**Antenatal corticosteroids (any)**	168 (59·6%)	148 (52·9%)
**Complete course received**	106 (37·6%)	106 (37·9%)
**Intensive care unit admission**	4 (1·4%)	10 (3·6%)
**Length of intensive care unit stay, nights**	2·5 (1·5, 3·0)<br>n=4	1·5 (1·0, 2·0)<br>n=10
**Obstetric high dependency unit admission**	51 (18·1%)	58 (20·7%)
**Length of high dependency unit stay, nights**	1·0 (1·0, 2·0)<br>n=51	1·0 (1·0, 3·0)<br>n=58
**Components by detected by a clinical diagnosis only (non-exclusive)**	**n=282**	**n=280**
Maternal death	1 (0·4%)	3 (1·1%)
Hepatic haematoma or rupture	0	0
Glasgow coma scale score <13	0	1 (0·4%)
Stroke	0	0
Cortical blindness	0	1 (0·4%)
Reversible ischaemic neurological deficit	0	0
Retinal detachment	0	1 (0·4%)
Postpartum haemorrhage requiring transfusion or hysterectomy	7 (2·5%)	10 (3·6%)
Placental abruption	3 (1·1%)	8 (2·9%)
Myocardial ischaemia/infarction	0	0
Eclampsia	3 (1·1%)	6 (2·1%)
Require >50% oxygen for greater than 1 h	2 (0·7%)	4 (1·4%)
Pulmonary oedema	0	2 (0·7%)
Severe breathing difficulty	0	3 (1·1%)
**Resource-dependent components**		
Hepatic dysfunction	30/171 (17·5%)	32/179 (17·9%)
Acute renal insufficiency	5/176 (2·8%)	5/190 (2·6%)
Dialysis	0	0
Transfusion of any blood product	28 (9·9%)	27 (9·6%)
Platelet count <50 x 10^9 per litre without blood transfusion	5/238 (2·1%)	4/250 (1·6%)

Positive inotropic support	0	3 (1·1%)
Intubation (other than for caesarean section)	0	2 (0·7%)
**Other secondary outcomes**		
**Magnesium sulfate: randomisation to delivery**	81 (28·7%)	96 (34·3%)
**Highest blood pressure recorded: randomisation to delivery**	**n=282**	**n=280**
Mean (SD) systolic blood pressure (mmHg)	152·75 (13·96)	157·03 (15·79)
Mean (SD) diastolic blood pressure (mmHg)	97·57 (10·13)	100·06 (10·82)
**Systolic blood pressure ≥160mmHg (randomisation to delivery)**	89 (31·6%)	121 (43·2%)
**Highest blood pressure recorded: delivery to post-delivery discharge**	**n=281**	**n=279**
Mean (SD) systolic blood pressure (mmHg)	149·72 (16·51)	148·73 (17·30)
Mean (SD) diastolic blood pressure (mmHg)	96·85 (12·17)	96·15 (12·47)
**Systolic blood pressure ≥ 160mmHg (delivery to post-delivery discharge)**	73 (26·0%)	73 (26·2%)
**Antihypertensive drugs administered (study entry to delivery)**	**n=282**	**n=280**
None	7 (2·5%)	6 (2·1%)
One oral agent	5 (1·8%)	12 (4·3%)
Two or more oral agents	270 (95·7%)	262 (93·6%)
One or more intravenous agent	39 (13·8%)	59 (21·1%)
**Antihypertensive drugs administered (non-exclusive)**		
Hydralazine	11 (3·9%)	26 (9·3%)
Labetalol	64 (22·7%)	79 (28·2%)
Methyldopa	175 (62·1%)	175 (62·5%)
Nifedipine	211 (74·8%)	211 (75·4%)
Atenolol	0	2 (0·7%)
Amlodipine	11 (3·9%)	16 (5·7%)
**Induction methods used**	**n=139**	**n=106**
Prostaglandin gel/pessary	27 (19·4%)	21 (19·8%)
Oral misoprostol	31 (22·3%)	31 (29·2%)
Vaginal misoprostol	57 (41·0%)	37 (34·9%)
Foley catheter	46 (33·1%)	35 (33·0%)
Artificial rupture of membranes	2 (7·2%)	2 (1·9%)
Oxytocin	10 (7·2%)	12 (11·3%)
Other (mifepristone)	6 (4·3%)	4 (3·8%)
	**n=282**	**n=280**
**Progression to HELLP syndrome**	1 (0·4%)	1 (0·4%)
**Estimated blood loss at delivery (mls)**	316 (212)<br>n=225	322 (209)<br>n=218
**Not measured**	57 (20·2%)	62 (22·1%)

HELLP syndrome - Haemolysis, Elevated liver enzymes, low platelets [defined as hepatic dysfunction and platelet count <50 x 10^9 per litre]. Data are n (%), mean (SD), or median (IQR).

Supplementary Table S3 Secondary descriptive perinatal outcomes

Outcome	Planned delivery n=301	Expectant management n=300
**Mode of birth**		
Spontaneous vaginal (cephalic)	112 (37·2%)	115 (38·3%)
Spontaneous vaginal (breech)	1 (0·3%)	0
Assisted vaginal (vacuum)	2 (0·7%)	4 (1·3%)
Assisted vaginal (forceps)	0	0
Assisted vaginal (breech)	0	0
Caesarean section	186 (61·8%)	181 (60·3%)
**Baby sex**		
Male	163 (54·2%)	151 (50·3%)
Female	138 (45·8%)	149 (49·7%)
**Gestation at birth**		
34 to <35 weeks	58 (19·3%)	30 (10·0%)
35 to <36 weeks	78 (25·9%)	82 (27·3%)
36 to <37 weeks	123 (40·9%)	88 (29·3%)
<37 weeks	259 (86·0%)	200 (66·7%)
≥37 weeks	42 (14·0%)	100 (33·3%)
**Principal recorded indication for neonatal unit admission (/infants admitted)**	**n=119**	**n=124**
Weight less than 1·8kg	18 (15·1%)	29 (23·4%)
In respiratory distress	22 (18·5%)	17 (13·7%)
Temperature >38 degrees Celsius	1 (0·8%)	0
Hypoglycaemia unresponsive to feeds	4 (3·4%)	0
Congenital anomalies	1 (0·8%)	2 (1·6%)
Asphyxia	19 (16·0%)	26 (21·0%)
Hypothermia	1 (0·8%)	0
Jaundice	7 (5·9%)	9 (7·3%)
Other	46 (38·7%)	40 (32·3%)
	**n=298**	**n=288**
**Apgar score at 10 minutes**	9·0 (9·0, 9·0) n=199	9·0 (9·0, 9·0) n=186
**Need for supplemental oxygen**	43/298 (14·4%)	55/288 (19·1%)
Days of supplemental oxygen required	2·79 (2·55) n=43	3·33 (5·27) n=55
**Antibiotics given for possible serious bacterial infection**	35 (11·7%)	34 (11·8%)
Number of days given	5·0 (3·0 to 7·0) n=35	7·0 (5·0 to 7·0) n=34
**Hypoxic ischaemic encephalopathy (HIE)**	14 (4·7%)	14 (4·9%)
Grade 1	7 (2·3%)	10 (3·5%)
Grade 2	6 (2·0%)	4 (1·4%)
Grade 3	1 (0·3%)	0
**Neonatal seizures requiring anticonvulsants**	3 (1·0%)	3 (1·0%)
**Administration of surfactant**	0	1 (0·3%)
**Hypothermia**	11 (3·7%)	8 (2·8%)
**Hypoglycaemia requiring intervention**	10 (3·4%)	9 (3·1%)
**Neonatal jaundice requiring phototherapy**	25 (8·4%)	27 (9·4%)
**Necrotising enterocolitis**	0	0

**Nasogastric feeding**	16 (5·4%)	20 (6·9%)
**Indication**	**n=16**	**n=20**
Prematurity	6 (37·5%)	8 (40·0%)
Infant on respiratory support	8 (50·0%)	10 (50·0%)
Hypoglycaemia	1 (6·3%)	2 (10·0%)
Phototherapy	1 (6·3%)	0
**Exclusively breast-fed at discharge**	279 (93·6%)	269 (93·4%)
**Number of infants admitted to each level of care**		
Acute care	2 (0·7%)	2 (0·7%)
Subacute care	90 (30·25)	104 (36·1%)
Kangaroo mother care	41 (13·8%)	42 (14·6%)
Normal care	243 (81·5%)	234 (81·3%)
**Number of nights in each level of care**	**n=298**	**n=288**
Acute care	7·50 (6·36)<br>n=2	1·50 (0·71)<br>n=2
Subacute care	4·68 (4·44)<br>n=90	4·91 (5·25)<br>n=104
Kangaroo mother care	4·68 (3·31)<br>n=41	4·48 (3·66)<br>n=42
Normal care	3·15 (1·98)<br>n=243	3·37 (2·61)<br>n=234
**Total number of nights in hospital**	4·68 (4·70)	5·18 (5·50)

Data are n (%), mean (SD), or median (IQR).

Supplementary Table S4 Serious Adverse Events

	Planned delivery n=282	Expectant management n=280
**Serious adverse events (SAEs)***	12 (4·2%)	21 (7·5%)
**Event**		
Pregnancy complicated by SAE	12 (4·2%)	20 (7·1%)
Maternal death	1 /12 (8·3%)	3/20 (15·0%)
Neonatal death prior to discharge	8/12 (66·7%)	6/20 (30·0%)
Antepartum stillbirth	0	10/20 (50·0%)
Intrapartum stillbirth	3/12 (25·0%)	2/20 (10·0%)
**Characteristics**	**n=12**	**n=21**
**Severity**		
Mild	0	0
Moderate	0	0
Severe	12 (100%)	21 (100%)
**Causality**		
Not related	9 (75·0%)	21 (100%)
Possibly	3 (25·0%)	0
Probably	0	0
**Action taken**		
Intervention stopped prior to the event starting	0	0
**Outcome**		
Fatal	12 (100%)	21 (100%)
Not resolved	0	0
Resolved	0	0
Resolved with sequelae	0	0

*There were 32 pregnancies complicated by 33 SAEs (one pregnancy was complicated by both an antepartum stillbirth and a maternal death).

Supplementary Table S5 Time from randomisation to initiation of delivery and delivery

	**Planned delivery n=282**	**Expectant management n=280**
**Time from randomisation to initiation of delivery, days**	2·37 (6·06)	5·54 (7·55)
**Time from randomisation to delivery, days**	3·01 (6·06)	5·89 (7·59)
**By gestational age at randomisation**		
**34^{+0} to 34^{+6}**		
n (%)	81 (28·7%)	78 (27·9%)
Days from randomisation to initiation of delivery	4·62 (9·71)	7·54 (10·48)
Days from randomisation to delivery	5·30 (9·54)	7·96 (10·51)
**35^{+0} to 35^{+6}**		
n (%)	83 (29·4%)	90 (32·1%)
Days from randomisation to initiation of delivery	2·04 (4·78)	5·91 (7·13)
Days from randomisation to delivery	2·73 (4·92)	6·20 (7·19)
**36^{+0} to 36^{+6}**		
n (%)	118 (41·8%)	112 (40.0%)
Days from randomisation to initiation of delivery	1·05 (1·60)	3·86 (4·53)
Days from randomisation to delivery	1·64 (1·83)	4·21 (4·57)

Data are n (%) or mean (SD).

Supplementary Table S6 Sensitivity analysis of women who had delivery initiated within 96 hours of randomisation

Outcome	Planned delivery	Expectant management	Risk Ratio* (95% CI)	P value
**Primary maternal outcome**	147/266 (55·3%)	168/280 (60·0%)	0·92 (0·80 to 1·06)	0·273
**Primary perinatal outcome**	53/285 (18·6%)	67/300 (22·35)	0·86 (0·62 to 1·20)	0·385

*Analysis adjusted for gestational age at randomisation, twin pregnancy, parity.

Supplementary Figure 1: Subgroup analysis

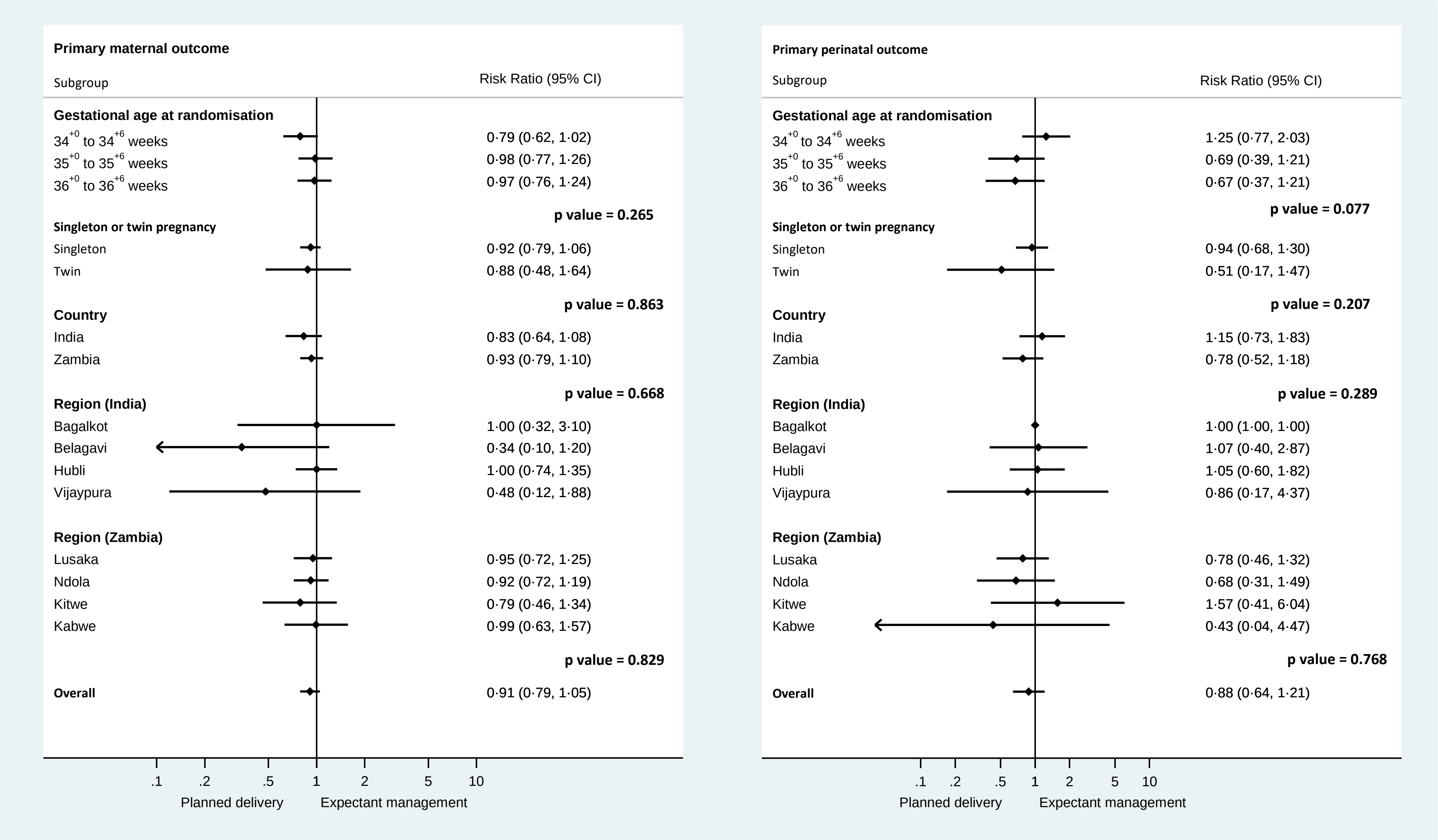

Supplement: Supplementary appendix [file mmc1.pdf]
